# Pharmacodynamic effects and plasma pharmacokinetics of *N*, *N-*dimethyltryptamine after intranasal versus subcutaneous administration in male rats

**DOI:** 10.1007/s00213-025-06879-8

**Published:** 2025-11-15

**Authors:** Michael H. Baumann, Grant C. Glatfelter, Sara E. Walton, Alex J. Krotulski, Christopher G. Witowski, Jacqueline L. von Salm

**Affiliations:** 1 Present Address: Designer Drug Research Unit, Intramural Research Program, National Institute on Drug Abuse, 333 Cassell Dr., Suite 4400, Baltimore, MD 21224 USA; 2 Center for Forensic Science Research and Education, Fredric Rieders Family Foundation, 206 Welsh Road, Horsham, PA 19044 USA; 3 College of Science and Technology, Temple University, 1801 Broad Street, Philadelphia, PA 19122 USA; 4 Psilera Inc, 3802 Spectrum Blvd., Suite 136B, Tampa, FL 33612 USA

**Keywords:** *N,N*-Dimethyltryptamine, Flat body posture, Hypothermia, Intranasal, Mass spectrometry, Pharmacokinetics, Psychedelic

## Abstract

**Rationale:**

There is growing interest in the therapeutic utility of psychedelic compounds that act as serotonin-2 A receptor (5-HT_2A_) agonists. *N*,*N*-dimethyltryptamine (DMT) is a 5-HT_2A_ agonist that produces intense and short-lived psychedelic subjective effects, but the compound requires non-oral routes of administration that bypass gastrointestinal metabolism.

**Objectives:**

Intranasal (IN) delivery of DMT represents one potential non-oral route of administration, but the feasibility of using this route is not well studied. Here, we examined the pharmacodynamic effects and plasma pharmacokinetics of DMT after IN and subcutaneous (SC) administration in rats.

**Methods:**

Male Sprague-Dawley rats fitted with intravenous (IV) catheters and SC temperature transponders received DMT fumarate (1, 3, or 10 mg/kg) or saline vehicle by IN or SC routes. Blood samples were withdrawn via catheters at various times after treatment, with behavioral scores and body temperatures measured prior to each blood draw. Plasma DMT and its *N*-oxide metabolite were quantified using liquid chromatography tandem quadrupole mass spectrometry (LC-QQQ-MS).

**Results:**

DMT produced a similar spectrum of pharmacodynamic effects after both routes, including increases in flat body posture and decreases in core body temperature. DMT displayed more rapid pharmacokinetics after the IN route (t_1/2_ range = 11.9–14.3 min) when compared to the SC route (t_1/2_ range = 45.5–122.7 min), and peak drug concentrations were greater with IN delivery.

**Conclusions:**

Our findings show the feasibility of using IN administration to deliver DMT in a reproducible and non-invasive manner. Importantly, the maximal DMT concentrations in rats given low IN doses (i.e., 30.2–55.6 ng/mL DMT) overlap with those reported in humans receiving psychoactive doses.

## Introduction

Over the past decade, there has been renewed interest in the therapeutic utility of psychedelic compounds that act as serotonin-2 A receptor (5-HT_2A_) agonists in the central nervous system (CNS) (Nichols et al. 2017; Garcia-Romeu and Richards 2018; Andersen et al. 2021). In particular, the mushroom constituent, 4-phosphoryloxy-*N*,* N*-dimethyltryptamine (psilocybin), is a psychedelic compound that shows efficacy as a pharmacological adjunct in treating depression, anxiety, and substance use disorders (Griffiths et al. 2016; Johnson et al. 2017; Carhart-Harris et al. 2021; Davis et al. 2021; Goodwin et al. 2022). Despite the positive outcomes with psilocybin, there are problems with its clinical use, including a sustained duration of psychedelic subjective effects lasting many hours. *N*,*N*-dimethyltryptamine (DMT) is a structural analog of psilocybin that produces rapid, intense, and short-lived psychedelic effects (Strassman et al. 1994; Carbonaro and Gatch 2016; Good et al. 2023; Vogt et al. 2023), suggesting DMT might be a useful alternative to psilocybin in some clinical contexts. In vitro pharmacological investigations show that DMT acts as an agonist at 5-HT_2A_, but the compound also has appreciable agonist activity at other 5-HT receptors, including the serotonin-1 A receptor (5-HT_1A_) (Rickli et al. 2016; Cameron and Olson 2018; Kozell et al. 2023).

Traditionally, DMT is the main psychoactive ingredient in Ayahuasca, a plant-based aqueous extract (i.e., tea or brew) that is used by indigenous populations in South America during religious ceremonies. Ayahuasca is made from a mixture of plant species enriched in DMT (e.g., *Psychotria* spp.) and β-carboline monoamine oxidase inhibitors (MAOIs) (e.g., *Banisteriopsis* spp.) (Domínguez-Clavé et al. 2016; Cameron and Olson 2018; James et al. 2022). Because DMT is rapidly degraded by monoamine oxidase enzymes in the gastrointestinal tract, orally ingested DMT requires co-administration of MAOIs to achieve pharmacological activity (Szára 1959; Riba et al. 2014; Barker 2022; Good et al. 2023). Lesser known plant-based concoctions containing various tryptamines (i.e., DMT, bufotenine, 5-methoxy-DMT, etc.) and MAOIs have been used in nasal delivery forms, like the snuff powders derived from the genus *Anadenanthera* or *Virola* (de Smet 1985; Ott 2001). The sacred rituals and practices of indigenous populations provide valuable insights into the therapeutic potential of natural drug mixtures, and Ayahuasca is reported to induce antidepressant effects similar to those produced by psilocybin in controlled human trials (see Sanches et al. 2016; Palhano-Fontes et al. 2019). Nevertheless, the scalability, reproducibility, and dependability of natural mixtures are not conducive to large-scale production via Good Manufacturing Practices (GMP), as required by the United States of America (USA) Food and Drug Administration (FDA). Established synthetic methods for purified DMT do exist (Cozzi and Daley 2020), but alternative pharmaceutical formulations are necessary for regulatory compliance and ultimately FDA approval in the current medical paradigm.

Because DMT, as a standalone drug, is not pharmacologically active after oral ingestion, other routes of administration, such as intravenous (IV), intramuscular, inhalation, insufflation, or transdermal, are required to reduce gastrointestinal first-pass effects (see Witowski et al. 2024). There is limited research into non-oral delivery methods of DMT, with only one early-stage clinical investigation using intranasal (IN) DMT administration after pretreatment with the MAOI, harmine (Dornbierer et al. 2023). Most human trials with DMT have used IV infusions, or combinations with MAOIs, at doses designed for maximum psychedelic subjective effects (0.2–0.4 mg/kg) (Strassman et al. 1994; Gallimore and Strassman 2016; Good et al. 2023; Vogt et al. 2023). Invasive delivery methods like IV administration pose some risks because indwelling catheters must be inserted and maintained for drug infusions. On the other hand, IV DMT infusions offer the advantage of rapidly adjusting the level of acute intoxication via careful dose titration (Erne et al. 2025). Formulations requiring MAOIs can create complications, since patient populations often display genetic variability in monoamine oxidase activities. For these reasons, there is great interest in investigating and developing alternative, non-invasive drug delivery routes for therapeutic dosing of DMT without MAOIs.

**Fig. 1:**
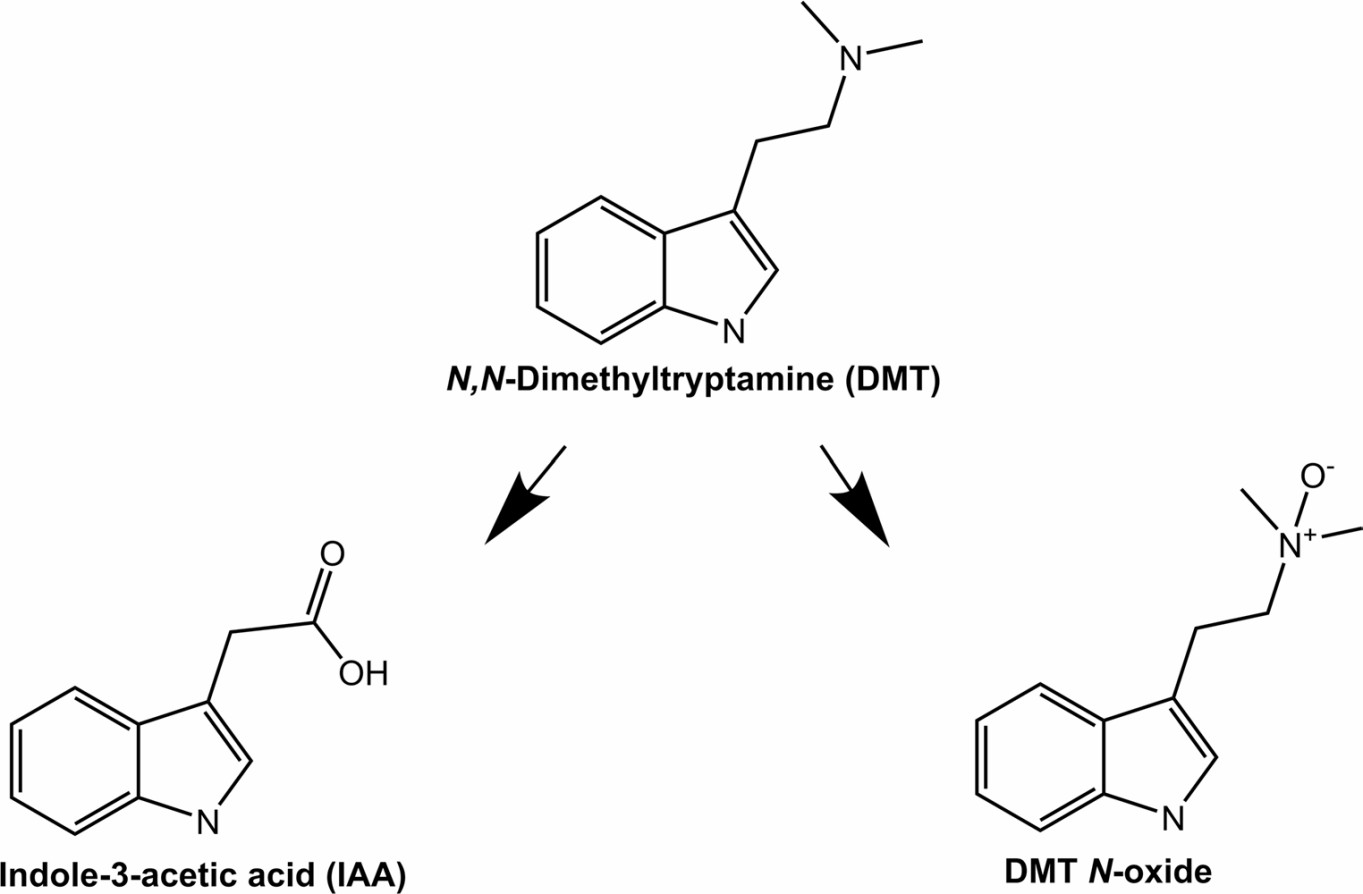
Chemical structures of *N*,* N*-dimethyltryptamine (DMT) and its metabolites, indole-3-acetic acid (IAA) and DMT *N*-oxide

In the present study, we examined the IN administration of DMT as a non-invasive method for drug delivery in male rats fitted with surgically-implanted IV catheters. Rats received various doses of DMT by the IN or subcutaneous (SC) route, and blood samples were collected via the catheters at various times thereafter. Plasma DMT and its *N*-oxide metabolite (DMT *N-*oxide) (see Fig. 1 for chemical structures) were quantified using liquid chromatography tandem quadrupole mass spectrometry (LC-QQQ-MS). We found that DMT displays more rapid pharmacokinetics after the IN route as compared to the SC route, and peak drug concentrations were greater with the IN route. In short, our findings provide proof-of-principle evidence for using the IN route of administration to deliver DMT in a reproducible, effective, and non-invasive manner.

## Materials and methods

### Drugs and reagents

For the animal experiments, DMT for injection was obtained from the NIDA Drug Supply Program as the fumarate salt (1:1 DMT : fumarate). For analytical methods, the reference standards for DMT, tryptamine, *N*-methyltryptamine (NMT), DMT *N*-oxide, 1,2,3,4-tetrahydrobetacarboline (THBC), 2-methyl-1,2,3,4-tetrahydrobetacarboline (2-methyl THBC), and *N*,* N*-dimethyltrypamine-D4 (DMT-D4) were purchased from Cayman Chemical (Ann Arbor, MI, USA). Our analytical method was developed to enable quantitation of DMT alone or in Ayahuasca-type mixtures, so reference standards consisted of potential DMT metabolites (e.g., DMT *N*-oxide) and ß-carboline constituents of Ayahuasca (e.g., THBC). It is noteworthy that the main DMT metabolite, indole-3-acetic acid (IAA), was not included in the quantitative scope because preliminary experiments demonstrated poor IAA recovery, precluding accurate quantitation.

Liquid chromatography mass spectrometry (LC-MS) grade water and methanol, as well as lab-grade dichloromethane and isopropanol, were obtained from VWR (Radnor, PA, USA). Sodium phosphate dibasic, sodium dihydrogen phosphate monohydrate, acetic acid, ammonia, and ammonium formate were purchased from Sigma-Aldrich (St. Louis, MO, USA). Formic acid ampules (1 mL) were obtained from Life Technologies Corporation (Carlsbad, CA, USA). Chromabond^®^ solid phase extraction cartridges were purchased from Thomas Scientific (Chadds Ford, PA, USA). Drug-free Sprague-Dawley pooled gender unspecified rat plasma preserved with dipotassium ethylenediaminetetraacetic acid (K_2_EDTA) was purchased from BioIVT (Westbury, NY, USA).

### Animals and surgery

Forty male Sprague-Dawley rats (250–300 g) purchased from Envigo (Frederick, MD, USA) were group housed (2–3 per cage) under conditions of controlled temperature (22 ± 2 °C) and humidity (45% ± 5%), with free access to food and water. The vivarium facilities were fully accredited by the Association for Assessment and Accreditation of Laboratory Animal Care. Lights were on between 7:00 a.m. and 7:00 p.m. The Institutional Animal Care and Use Committee of the National Institute on Drug Abuse (NIDA), Intramural Research Program (IRP), approved the animal experiments, and all procedures were carried out in accordance with the approved NIDA IRP Animal Study Proposal 23-OSD-37.

Rats were anesthetized with intraperitoneal (IP) ketamine and xylazine (75 and 5 mg/kg, respectively) (Covetrus, Dublin, OH, USA), and IV catheters were surgically implanted into the right jugular vein. Catheters were constructed of Silastic medical grade tubing (Dow Corning, Midland, MI, USA) linked to vinyl tubing (SCI, Lake Havasu City, AZ, USA) using a 23 g stainless steel connector. In brief, the proximal Silastic end of the catheter was advanced to the atrium, while the distal vinyl end was exteriorized on the nape of the neck and plugged with a metal stylet. Immediately after catheter implantation, while rats were still under anesthesia, temperature transponders (model IPTT-300; Bio Medic Data Systems, Seaford, DE, USA) were surgically implanted to allow for non-invasive measurement of body temperature (Walton et al. 2023). The temperature transponders emit radio frequency signals received by a compatible handheld reader system (DAS-7006/7r; Bio Medic Data Systems). The transponders are cylindrical in shape (14 × 2 mm) and were implanted SC along the midline of the back, posterior to the shoulder blades, via a prepackaged sterile guide needle delivery system. Rats were single housed postoperatively and given three to seven days to recover from surgery.

### Animal experiments

Rats were randomly assigned to groups receiving either IN (*n* = 20 rats) or SC (*n* = 20 rats) treatments. Each rat was used in only one experiment, and received a single treatment. On the day of an experiment, rats were brought into the laboratory in their home cages and allowed 1 h to acclimate to the surroundings. Food and water were removed from the cages, and micro-isolator lids were replaced with wire cage tops. Polyethylene extension tubes were attached to 1 mL tuberculin syringes and filled with sterile saline before being connected to the vinyl end of the IV catheters. Blood sampling, performed by an investigator remote from the animal, was facilitated by threading the extension tubes through the wire cage tops to the outside of the cages. Catheters were flushed with 0.3 mL of 48 IU/mL heparin saline (Thomas Scientific, Swedesboro, NJ, USA) to facilitate blood withdrawal.

DMT for IN injection was prepared in sterile 0.9% saline at concentrations of 10, 30, and 100 mg/mL, which were delivered in a volume of 0.1 mL/kg to yield administered doses of 1, 3, and 10 mg/kg. For IN injections, rats were briefly anesthetized with isoflurane (Covetrus) using a drop-jar method under a fume hood. Once immobilized (15–30 s), DMT or vehicle solutions were delivered IN using a 100 µL Hamilton microsyringe (Reno, NV, USA), with half of the volume infused into the right nares and the other half into the left nares. Total injection volumes ranged from 35 to 40 µL. DMT for SC injection was prepared in sterile 0.9% saline at concentrations of 1, 3, and 10 mg/mL, which were delivered in a volume of 1 mL/kg to yield administered doses of 1, 3, and 10 mg/kg. All injected DMT doses are expressed as the weight of the 1:1 fumarate salt.

For the IN DMT experiments, blood samples (300 µL) were withdrawn via IV catheters immediately before (time zero) and at 5, 10, 20, 40, and 80 min after injection. For the SC DMT experiments, blood samples were withdrawn immediately before (time zero) and at 10, 20, 40, 80, and 160 min after injection. Samples were collected into 1 mL tuberculin syringes and transferred to 1.5 mL plastic tubes containing 5 µL of 1000 IU/mL heparin (Thomas Scientific) as an anticoagulant and 5 µL of 250 mM sodium metabisulfite as a preservative. Blood was centrifuged at 1000 *g* for 10 min at 4 °C. Plasma was decanted into cryovials and stored at − 80 °C until analysis. To maintain volume and osmotic homeostasis, rats received an equal volume of saline solution via the IV catheter after each blood withdrawal.

Prior to each blood withdrawal, specific behaviors were scored, and body temperature was measured. The behaviors of interest included motor activity (i.e., ambulation in the horizontal plane), flat body posture (i.e., an element of the 5-HT syndrome) (Tricklebank et al. 1985; O’Connell and Curzon 1996), and wet dog shakes (i.e., a proxy for 5-HT_2A_ activation) (Buchborn et al. 2015; Elmore et al. 2018). Rat behaviors were observed by an experienced rater for 1 min prior to blood withdrawal, and the occurrence of motor activity and flat body posture was numerically scored as either absent = 1 or present = 2. The total number of wet dog shakes over the 1-min period was recorded. Immediately after behavioral observations, body temperature was measured non-invasively by placing the handheld reader in the vicinity of the implanted transponder on the back of the rat.

### Analytical methods

Samples were extracted using a basic solid phase extraction (SPE) procedure. Spiking solutions (final concentrations 0.1, 1.0 and 10 ng/µL) contained all analytes and were prepared by serial dilution in methanol from stock solutions. Calibration curves contained eight non-zero calibrators from 5 to 1000 ng/mL. Quality controls were assessed at 15, 60, 150, and 800 ng/mL. DMT-D4 (25 µL, 1.0 ng/µL) was utilized as the internal standard. Authentic rat plasma samples were stored at −80 °C prior to analysis.

Drug-free rat plasma was fortified with appropriate spiking solutions and aliquoted (150 µL) for extraction. Authentic rat plasma was assayed at 150 µL. Internal standard was added at a final concentration of 125 ng/mL. Samples were diluted with 1 mL phosphate buffer (0.1 M, pH 6) followed by centrifugation for 5 min at 4200 rpm, then extracted using Chromabond^®^ SPE cartridges (200 mg, 3 mL)(Macherey-Nagel, Allentown, PA, USA). The columns were conditioned using methanol (2 mL), deionized water (2 mL), and phosphate buffer (0.1 M, pH 6, 2 mL). The buffered sample was added to the columns and impurities were removed using deionized water (1 mL), acetic acid (0.1 M, 1 mL), and methanol (1 mL). Analytes were eluted using 3 mL of dichloromethane: isopropanol: ammonia (78:20:2, *v: v:v*). The organic solvent was evaporated to dryness under nitrogen at 33 °C. Samples were reconstituted in 100 µL of initial mobile phase conditions (90 A:10B).

Analysis was performed via LC-QQQ-MS using a Waters Xevo TQ-S Micro coupled with a Waters Acquity UPLC^®^ (Waters Millipore, Milford, MA, USA). Chromatographic separation was achieved using a Waters Acquity UPLC^®^ HSS C-18 analytical column (2.1 × 100 mm, 1.8 μm). The column temperature was 60 °C with a flow rate of 0.4 mL/min. The injection volume was 10 µL. The mobile phase compositions were 5 mM ammonium formate in water, pH 3 (A) and 0.1% formic acid in methanol (B). Total assay run time was 5.5 min with the following gradient: 10% B initially, increasing to 60% B over 4.0 min, at 4.1 min ramping to 95% B for an organic flush for 0.5 min, and returning to initial conditions at 4.7 min with a 0.8 min hold. The instrument was operated using positive ion electrospray (ESI+). The desolvation gas temperature was at 500 °C at 1000 L/h with a source temperature of 150 °C. The voltage for the capillary was 0.5 kV. Multiple reaction monitoring (MRM) transitions and analyte specific mass spectrometer settings are in Table 1.

**Table 1:** Retention times, MRM transitions, and mass spectral parameters

Compound	Retention Time (min)	Precursor Ion (Da)	Product Ions (Da)	Dwell Time (s)	Cone (V)	Collision (V)
Tryptamine	2.29	173.1	91.1 117.1* 127.1	0.0010	20	24 20 18
DMT	2.32	189.1	58.1* 91.1 117.1	0.020	18	8 24 20
NMT	2.37	175.1	91.1 117.1 144.1*	0.020	18	22 18 10
DMT N-Oxide	2.68	205.1	117.1 127.1 144.1*	0.020	18	20 22 12
2-methyl THBC	2.72	187.1	77.1* 117.1 128.1	0.020	18	36 28 32
THBC	2.77	173.1	77.1 127.1 144.1*	0.0010	20	34 26 15
DMT-D4	2.30	193.1	60.1* 118.1 148.1	0.020	24	12 38 30

*Quant ion

The method was validated following the standards issued by the American Academy of Forensic Science (AAFS) Standards Board (ASB): Standard Practices for Method Validation in Forensic Toxicology (ASB, 2019). The following parameters were assessed: calibration model, limit of detection (LOD), limit of quantitation (LOQ), precision, bias, accuracy, recovery, dilution, integrity, carryover, determination of interferences from matrix, analyte, internal standard, and commonly encountered drugs (*n* = 264)

### Statistical analyses

Behavioral data were analyzed using one-way Welch’s ANOVA (dose) followed by Dunnett’s T3 post hoc test, whereby the summed behavioral scores for drug-induced motor activity and flat body posture, as well as total number of wet dog shakes, were compared to vehicle controls. The time course data for body temperature, plasma concentrations of DMT, and plasma concentrations of DMT *N-*oxide were analyzed by two-way ANOVA (dose x time) followed by Tukey’s multiple comparison test. The time-concentration profiles for plasma DMT were further evaluated using non-compartmental pharmacokinetic modeling (Kinetica, Thermo Fisher Scientific, Waltham, MA, USA) to determine parameters including maximal concentration (C_max_), time of maximal concentration (t_max_), plasma half-life (t_1/2_), and area-under-the-curve (AUC). The calculated pharmacokinetic parameters were subjected to one-way ANOVA (dose) followed by Tukey’s multiple comparisons test. To investigate pharmacokinetic/pharmacodynamic relationships, mean plasma DMT concentrations from individual rats were compared to mean DMT *N-*oxide concentrations, summed flat body scores, and mean body temperature values using Spearman’s correlation test. The minimum criterion for statistical significance was *p* < 0.05 in all cases.

## Results

### Pharmacodynamic Effects of DMT

**Fig. 2:**
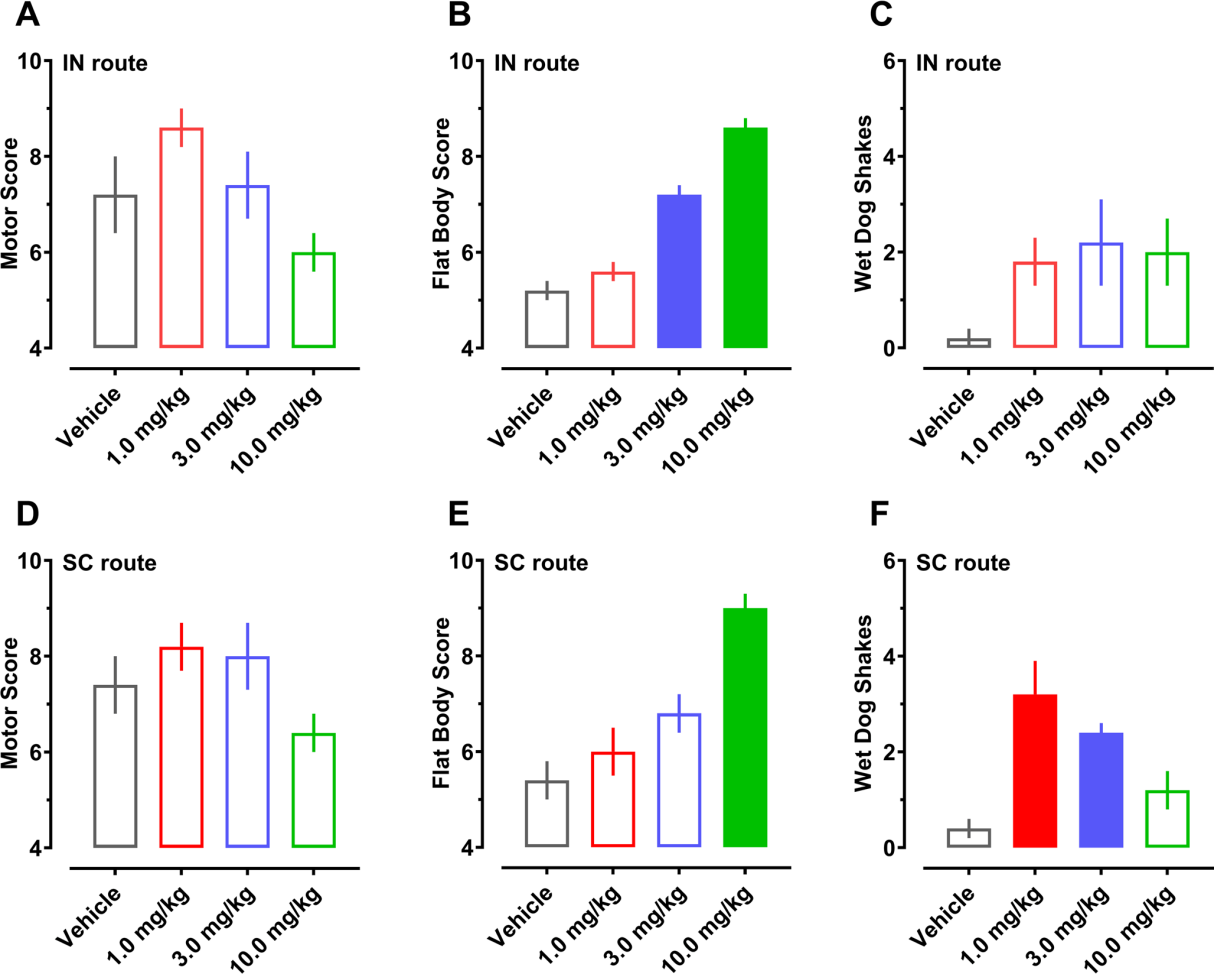
Effects of intranasal (IN) or subcutaneous (SC) DMT administration on behaviors in male Sprague-Dawley rats. Summed motor scores (**A**&**D**), summed flat body scores (**B**&**E**), and total wet dog shakes (**C**&**F**) were determined after DMT administration, as described in Materials and Methods. Data are mean ± SEM for *n* = 5 rats/group. Filled bars represent significant differences compared to vehicle groups (*p*<0.05)

Figure 2 depicts the effects of IN and SC DMT administration on various behaviors in male rats undergoing serial blood sampling. Figure 2 A**&D** illustrate the effects of DMT on summed motor scores over the course of the sessions. In general, DMT slightly increased motor activity after 1 mg/kg but decreased activity after 10 mg/kg. Welch’s ANOVA showed a significant main effect of DMT dose on motor scores after IN (W = 6.106[3.000, 8.596], *p* = 0.0162), but not SC (W = 2.695[3.000, 8.715], *p* = 0.1108) administration. However, Dunnett’s post hoc tests revealed no significant differences in drug-induced motor scores across doses, when compared to vehicle controls, for either route of administration. Figure 2B**&E** demonstrate that DMT significantly impacted flat body scores after IN (W = 52.97[3.000, 8.889], *p* < 0.0001) and SC (W = 18.20[3.000, 8.751], *p* = 0.0004) administration. Flat body posture is an element of the 5-HT behavioral syndrome in rats that is thought to involve activation of centrally-located 5-HT_1A_ (Tricklebank et al. 1984; O’Connell and Curzon 1996). For IN DMT, flat body posture was increased above vehicle at 3 and 10 mg/kg, whereas for SC DMT, flat body posture was increased only at the 10 mg/kg dose. Figure 2C**&F** show the effects of DMT on total number of wet dog shakes, a behavioral index of 5-HT_2A_ activation in the rat (Buchborn et al. 2015; Elmore et al. 2018). Welch’s ANOVA showed a significant main effect of dose on wet dog shakes after IN (W = 4.800[3.000, 7.631], *p* = 0.0359) and SC (W = 16.33[3.000, 8.401], *p* = 0.0007) administration. However, post hoc tests revealed significant differences only in the SC dosing group, where wet dog shakes were increased compared to vehicle after 1 and 3 mg/kg.

**Fig. 3:**
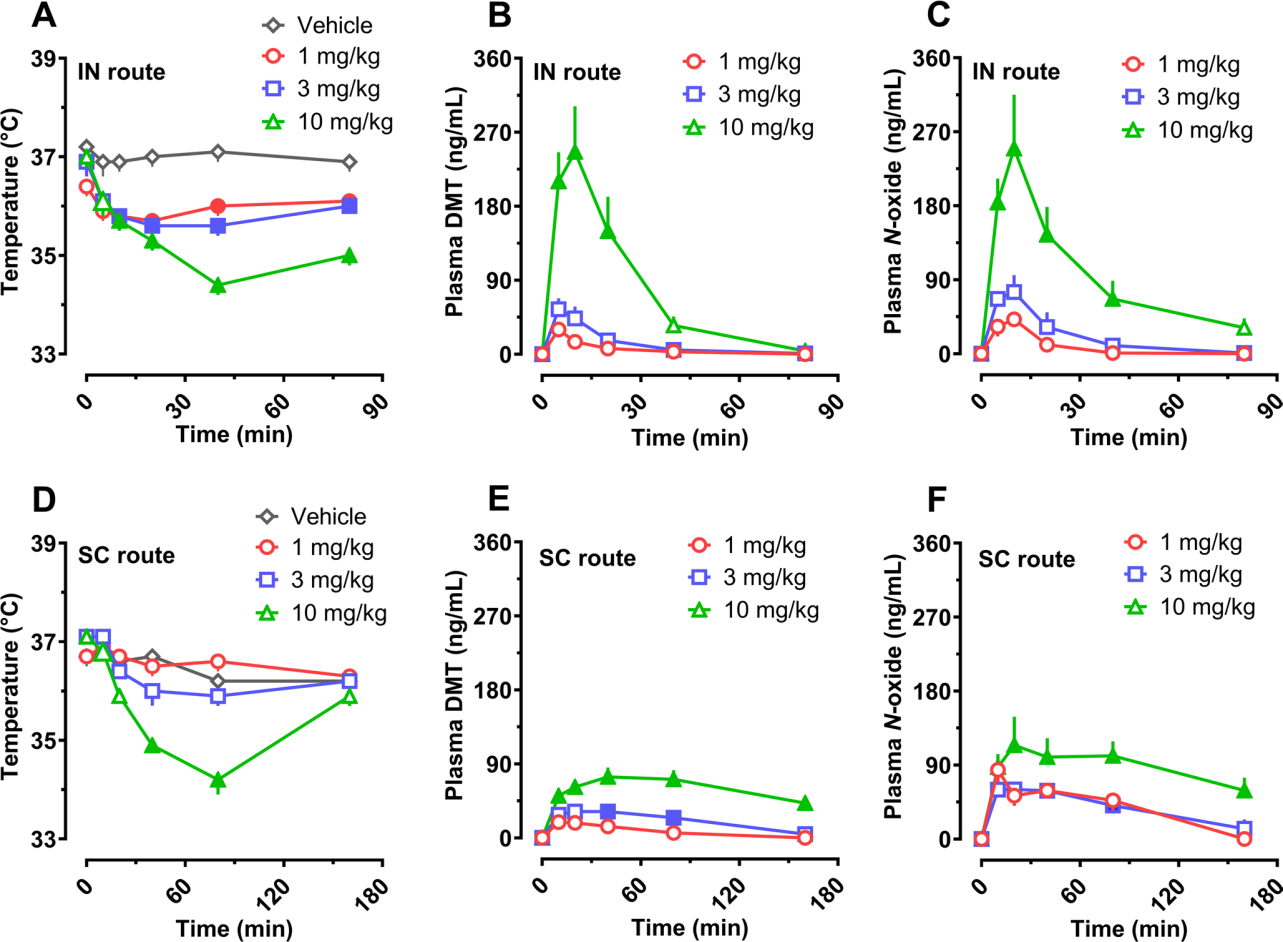
Time-course effects of intranasal (IN) or subcutaneous (SC) DMT administration on body temperature (**A**&**D**) and plasma concentrations of DMT (**B**&**E**) and DMT *N*-oxide (**C**&**F**). Body temperature measures and blood samples were obtained at various times after IN or SC DMT, as described in Materials and Methods. Plasma DMT and DMT *N*-oxide were quantitated by LC-QQQ-MS. Data are mean ± SEM for *n* = 5 rats/group. For temperature data, filled symbols represent significant differences compared to the vehicle group at a specific time point. For plasma analyte concentrations, filled symbols represent significant differences compared to the 1 mg/kg dose group at a specific time point

Figure 3 depicts the time-course effects of DMT on core body temperature and plasma concentrations of DMT and its metabolite, DMT *N*-oxide. Figure 3A&D shows the time-course effects of DMT on body temperature. For IN DMT administration, two-way ANOVA found significant main effects of dose (F[3, 96] = 49.99, *p* < 0.0001) and time (F[5, 96] = 14.10, *p* < 0.0001), along with a significant dose x time interaction (F[15, 96] = 3.952, *p* < 0.0001). Post hoc tests revealed that IN DMT at all doses significantly reduced temperature compared to saline controls by 20 min post-treatment. The hypothermic effect of 10 mg/kg IN DMT reached a nadir of 34.4 °C and was sustained throughout the sampling period (i.e., 80 min). For SC DMT administration, two-way ANOVA found significant main effects of dose (F[3, 96] = 23.02, *p* < 0.0001) and time (F[5, 96] = 25.24, *p* < 0.0001), along with a significant dose x time interaction (F[15, 96] = 6.403, *p* < 0.0001). In the case of SC DMT, only the 10 mg/kg dose significantly reduced temperature compared to vehicle control; this hypothermic effect reached a nadir of 34.2 °C, but effects subsided by the end of the sampling period (i.e., 160 min).

### Analytical method validation

The analytical method validation met or exceeded ASB acceptability criteria. All analytes were determined to have a quadratic calibration model with a 1/X^2^ weighting and produced coefficients of determination greater than or equal to 0.98. Recovery was greater than 87% for all analytes except for DMT *N*-oxide (51%); however, no impacts on quantitation were observed. The LOQs for DMT and 2-methyl THBC were 5 ng/mL, while the LODs were 2 ng/mL and 5 ng/mL, respectively. The LOQs for NMT, DMT *N*-oxide, THBC, and tryptamine were 20 ng/mL, while the LODs were 2, 20, 20, and 20 ng/mL, respectively. No interferences were detected from matrix, analyte, internal standard, or common drugs (*n* = 264). Carryover was evaluated at twice the high calibrator (2000 ng/mL) and no carryover was observed.

### Pharmacokinetics of DMT and DMT ***N***-oxide

Figure 3B&E show the time-concentration profiles for plasma DMT after IN or SC treatment. For IN administration, there was a main effect of DMT dose (F[2, 72] = 46.80, *p* < 0.0001) and time (F[5, 72] = 17.84, *p* < 0.0001), along with a significant dose x time interaction (F[10, 72] = 7.899, *p* < 0.0001). DMT concentrations after the IN route were generally dose-proportional, though analyte concentrations appeared higher than predicted after the 10 mg/kg IN dose (see DMT AUC data below). Tukey’s post hoc test revealed that DMT concentrations after 10 mg/kg IN were significantly greater at 5, 10, and 20 min post-treatment when compared to those after 1 and 3 mg/kg IN. After all doses of IN DMT, the plasma concentrations of DMT were below the LOD, or nearly so, by 80 min post-treatment. For SC administration, there was a main effect of DMT dose (F[2, 72] = 132.7, *p* < 0.0001) and time (F[5, 72] = 36.71, *p* < 0.0001), along with a significant dose x time interaction (F[10, 72] = 7.414, *p* < 0.0001). DMT concentrations after the SC route were dose-proportional, and post hoc tests revealed that concentrations after 3 mg/kg SC DMT were significantly greater than those after 1 mg/kg at 20 and 40 min post-treatment. At the 10 mg/kg SC dose, plasma DMT concentrations were greater than those after 1 mg/kg SC at all time points post-injection.

Figure 3C&F show the time-concentration profiles for plasma DMT *N-*oxide after IN or SC administration of DMT. In general, the time-course for circulating DMT *N-*oxide tracked with that of the parent compound for both routes of administration. After IN DMT, there was a main effect of dose (F[2, 72] = 36.86, *p* < 0.0001) and time (F[5, 72] = 15.85, *p* < 0.0001) on DMT *N-*oxide, along with a significant dose x time interaction (F[10, 72] = 3.918, *p* = 0.0003). DMT *N*-oxide concentrations after the IN route were generally dose-proportional. Tukey’s post hoc tests revealed that DMT *N-*oxide concentrations after 10 mg/kg IN DMT were significantly greater at 5, 10, 20, and 40 min post-treatment when compared to those after 1 and 3 mg/kg IN DMT. After all doses of IN DMT, the concentrations of DMT *N-*oxide were either undetectable or very low by 80 min post-treatment. After SC administration of DMT, there was a main effect of dose (F[2, 72] = 14.81, *p* < 0.0001) and time (F[5, 72] = 16.68, *p* < 0.0001) on DMT *N*-oxide, but there was no significant dose x time interaction (F[10, 72] = 1.248, *p* = 0.2761). DMT *N-*oxide concentrations after the SC route were not proportional at low doses, with nearly identical *N-*oxide concentrations after the 1 and 3 mg/kg treatments. Post hoc tests revealed that concentrations of DMT *N*-oxide were greater after 10 mg/kg SC DMT when compared to concentrations after 1 and 3 mg/kg doses of SC DMT.

The time-concentration profiles for DMT were subjected to non-compartmental pharmacokinetic analysis (Kinetica, Thermo Fisher) to calculate C_max_, t_max_, t_1/2_, and AUC values as summarized in Table 2. For IN DMT administration, the C_max_ rose significantly with increasing dose (F[2, 12] = 17.85, *p* = 0.0003), and C_max_ values after 10 mg/kg were significantly greater than those after 1 and 3 mg/kg doses. By contrast, there were no significant differences in DMT t_max_ (F[2, 12] = 1.132, *p* = 0.3545) or t_1/2_ values (F[2, 12] = 0.3251, *p* = 0.7286) across different IN doses, and drug half-lives ranged from 11.9 to 14.3 min. Similar to the C_max_ findings for IN DMT, AUC values rose significantly with increasing IN dose (F[2, 12] = 13.34, *p* = 0.0009), and AUC values after 10 mg/kg were significantly elevated compared to AUC values after 1 and 3 mg/kg doses. The AUC measures after IN DMT provided evidence for non-linear accumulation of the drug at the 10 mg/kg dose. Specifically, the predicted AUC after 10 mg/kg IN DMT is 2950 min*ng/mL (i.e., ~ 3.3-times the measured AUC at 3 mg/kg), whereas the actual measured AUC was much higher at 5626 min*ng/mL. For SC DMT administration, C_max_ rose significantly with increasing dose (F[2, 12] = 14.20, *p* = 0.0007), and C_max_ values after 10 mg/kg were greater than those after 1 and 3 mg/kg doses. The t_max_ after SC DMT was significantly impacted by dose (F[2, 12] = 17.49, *p* = 0.0003), with delayed t_max_ values as dose increased. In particular, t_max_ after 10 mg/kg SC DMT was significantly delayed compared to that after 1 mg/kg. In agreement with the t_max_ findings, there was a tendency for t_1/2_ measures to increase with higher DMT doses, but this effect did not reach statistical significance (F[2, 12] = 2.108, *p* = 0.1642). After SC DMT, AUC values rose with increasing dose (F[2, 12] = 40.72, *p* < 0.0001), and AUC values after 10 mg/kg were significantly elevated compared to 1 and 3 mg/kg doses.

**Table 2:** DMT Pharmacokinetic parameters calculated from time-concentration data depicted in Fig. 3

DMT Dose	DMT Route	C_max_ (ng/mL)	t_max_ (min)	t_1/2_ (min)	AUC (min*ng/mL)
1 mg/kg	IN	30.1 ± 7.6	5.0 ± 0.0	12.9 ± 2.8	365 ± 42
3 mg/kg	IN	55.6 ± 15.4	6.0 ± 1.1	14.3 ± 1.9	894 ± 260
10 mg/kg	IN	268.0 ± 50.7*	7.0 ± 1.2	11.9 ± 1.4	5626 ± 1348*
1 mg/kg	SC	19.6 ± 2.2	14.0 ± 2.4	45.5 ± 9.5	983 ± 72
3 mg/kg	SC	35.8 ± 2.4	22.0 ± 4.9	91.7 ± 26.4	2863 ± 228
10 mg/kg	SC	81.6 ± 11.1*	64.0 ± 9.8*	122.7 ± 36.4	9465 ± 1185*

Time course data were subjected to non-compartmental pharmacokinetic analysis, and data are mean ± SEM for *n* = 5 rats/dose group. Asterisks represent significant differences when compared to the 1 mg/kg DMT dosing group for a particular route of administration

### Pharmacokinetic/pharmacodynamic relationships

**Fig. 4:**
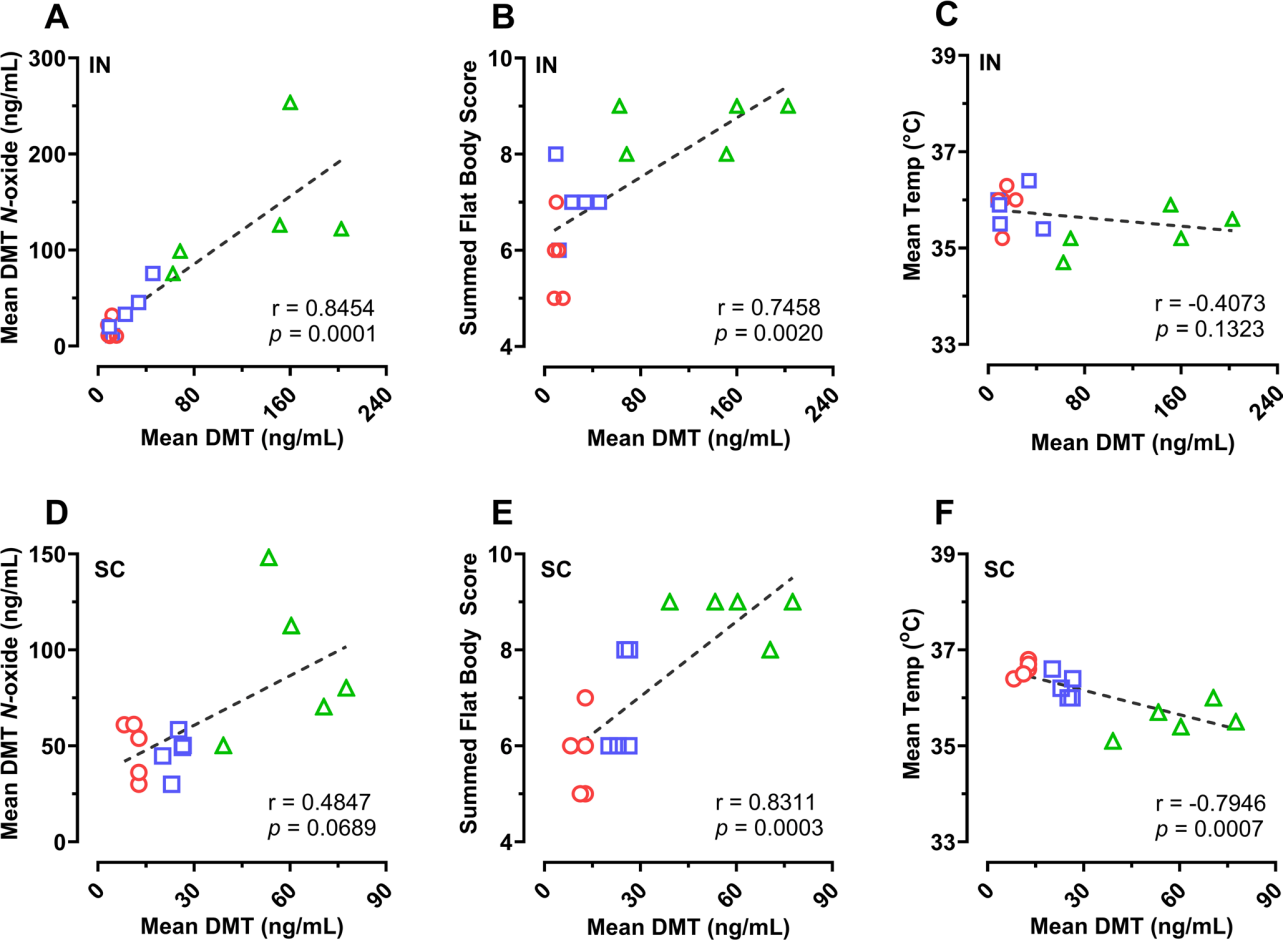
Correlation plots showing mean DMT concentrations versus mean DMT *N*-oxide concentrations (**A**&**D**), summed flat body scores (**B**&**E**), or mean temperatures (**C**&**F**) from rats receiving IN or SC *(n =* 15 total/route) DMT. The data points represent results from individual rats in each dose group (red circles are 1 mg/kg, blue squares are 3 mg/kg, and green triangles are 10 mg/kg). Mean concentrations of DMT, mean concentrations of DMT *N-*oxide, and mean body temperatures were determined from data shown in Fig. 3, whereas summed flat body scores were determined from data shown in Fig. 2

An additional aim of our study was to examine relationships between circulating concentrations of DMT, its metabolites, and pharmacodynamic effects. To this end, we carried out Spearman correlation analyses using mean DMT concentrations versus mean DMT *N*-oxide concentrations, summed flat body posture scores, and mean body temperatures from individual rats receiving either IN or SC *(n =* 15 rats/route) DMT. The correlation data are summarized in Fig. 4. For IN DMT administration, mean DMT concentrations were positively correlated with concentrations of the *N-*oxide metabolite (Fig. 4A) and summed flat body posture scores (Fig. 4B), whereas the relationship between DMT and mean temperature was not significant (Fig. 4C). For SC DMT administration, DMT concentrations were not significantly correlated with concentrations of *N*-oxide (Fig. 4D), but were positively correlated with summed flat body posture scores (Fig. 4E) and negatively correlated with mean temperature measures (Fig. 4F).

## Discussion

DMT is a serotonergic psychedelic that induces intense and short-lived psychedelic experiences in human subjects (Strassman et al. 1994; Carbonaro and Gatch 2016; Good et al. 2023; Vogt et al. 2023). Although DMT has garnered interest as a possible therapeutic agent (see D’Souza et al. 2022), the drug requires non-oral routes of administration due to its rapid metabolism by MAO enzymes in the gastrointestinal tract (Szára 1959; Riba et al. 2014; Barker 2022; Good et al. 2023). The anatomical location of the intranasal cavity facilitates direct access to the vasculature of the brain, increasing the bioavailability of CNS drugs with diverse chemical structures and indications (Chapman et al. 2012). For example, the clinically-approved dissociative drug Spravato^®^ (esketamine) employs the IN route of administration, whereas the psychedelic compound 5-methoxy-*N*,* N-*dimethyltryptamine (5-MeO-DMT) has an IN formulation that is currently in phase II development (Rucker et al. 2024). Both of the aforementioned drugs are characterized by the lack of oral bioavailability, and have routinely been administered via the IV route before the availability of IN formulations. As DMT possesses psychomimetic effects and therapeutic potential analogous to esketamine and 5-MeO-DMT, the IN delivery route could enhance patient compliance and clinical applications of DMT.

Here, we examined the pharmacodynamic effects and pharmacokinetics of DMT administered by the IN and SC routes in male Sprague-Dawley rats. We found that DMT causes similar pharmacodynamic effects after both routes of administration, including increases in flat body posture and wet dog shakes, along with a decrease in body temperature. DMT pharmacokinetics were generally dose-proportional after both routes of administration, but DMT displayed a much faster clearance after IN versus SC administration. DMT C_max_ values were generally higher with the IN route. DMT *N*-oxide concentrations tracked with the parent compound and reached similar absolute concentrations in plasma. Our correlation analyses demonstrate that plasma DMT concentrations generally predict pharmacodynamic effects after either route of administration, though there was no significant relationship between plasma DMT and hypothermia after the IN route.

We found that DMT produced a spectrum of behavioral effects in rats that was characterized by flat body posture and wet dog shakes. Both routes of DMT administration produced dose-related increases in flat body posture, whereas effects on wet dog shakes were more variable, with dose-related effects only after SC dosing. Few prior studies have examined non-contingent behavioral effects of DMT in rats, but our present findings agree with reported effects of ring-substituted DMT analogs, like 5-MeO-DMT (see Tricklebank et al. 1985; Halberstadt and Geyer 2011). Lucki et al. (1984) first demonstrated that intraperitoneal (IP) administration of 5-MeO-DMT to rats produces the 5-HT behavioral syndrome, a constellation of symptoms including side-to-side head weaving, forepaw treading, and flattened body posture. Subsequent research showed that the 5-HT syndrome produced by 5-MeO-DMT, and related tryptamines, is likely mediated via activation of post-synaptic 5-HT_1A_ in the CNS (Smith and Peroutka 1986; Tricklebank et al. 1985). In agreement with the older literature, more recent findings demonstrate that SC 4-hydroxy-*N*,* N*-dimethyltryptamine (psilocin) causes flat body posture in male and female rats that is qualitatively similar to what we observed here with DMT (Tylš et al. 2016).

DMT caused a modest increase in wet dog shakes after both routes of administration, but this effect was only significant for the SC route, where lower doses of DMT produced the most wet dog shakes. The apparent route-specific difference in wet dog shakes might be explained by greater variance in the data obtained with the IN route. Additionally, the rats receiving IN infusions were briefly immobilized by isoflurane anesthesia and gently restrained, which could have altered the behavioral responsiveness to DMT. It is also worth noting that the frequency of wet dog shakes produced by DMT in the present work was much lower than the frequency of this behavior induced by phenylethylamine psychedelics like 1-(4-iodo-2,5-dimethoxyphenyl)propan-2-amine (DOI) in rats (e.g., see Baumann and Rothman 1996; Elmore et al. 2018). Previous experiments from our laboratory, using similar time-sampling methods of behavioral scoring, show SC DOI and related phenethylamines like 2-(4-iodo-2,5-dimethoxyphenyl)ethanamine (2 C-I) produce dose-related increases in wet dog shakes that culminate in more than ten episodes per hour. One possible explanation for the reduced number of wet dog shakes induced by DMT could be related to its preferential 5-HT_1A_ agonist effects (Rickli et al. 2016), a property which tends to reduce the expression of 5-HT_2A_-mediated behaviors in both rats and mice (Halberstadt 2015; Glatfelter et al. 2024). Clearly, more research is needed to further characterize the non-contingent behavioral effects of DMT in rats, and to determine the receptor mechanisms underlying these effects.

We found that DMT administration by either route caused decreases in body temperature, but hypothermic effects were dose-related only after SC DMT administration. With the IN route, all doses of DMT induced a rapid decline in temperature, which was sustained for the entire blood sampling session, even after circulating concentrations of DMT had fallen to undetectable levels. One possible reason for the sustained hypothermic effects of IN DMT could be the brief isoflurane exposure in those rats, which might alter the temperature response to subsequent drugs. On the other hand, rats treated with IN saline infusions exhibited no evidence for hypothermia, suggesting that brief isoflurane exposure per se does not reduce body temperature. In any case, our temperature results with DMT support the known hypothermic effects of DMT and its ring-substituted analogs in rats and mice (Winter 1972; Gudelsky et al. 1986; Glatfelter et al. 2022). Winter (1972) first noted the hypothermic effects of DMT in rats, and subsequent work demonstrated that hypothermia induced by 5-MeO-DMT, and related tryptamines, is mediated by 5-HT_1A_ in the CNS (Gudelsky et al. 1986; Nash et al. 1989; Glatfelter et al. 2022). It is worth mentioning that hypothermic effects of DMT noted here in rats differ from the slight hyperthermic effects produced by psychoactive doses of DMT in humans (Strassman and Qualls 1994).

A main focus of the present study was to compare the pharmacokinetics of DMT after IN versus SC administration. As expected, we found that IN administration engendered more rapid kinetics when compared to SC administration. The t_1/2_ values for IN DMT ranged from 11.9 to 14.3 min, whereas the t_1/2_ values for SC DMT ranged from 45.5 to 122.7 min, with delayed drug clearance as SC dose increased. DMT C_max_ values were higher after IN administration, and the AUC findings after IN dosing provided evidence for non-linear accumulation of the drug at the 10 mg/kg dose. Nevertheless, despite high C_max_ and AUC values after IN DMT, t_1/2_ estimates were remarkably consistent across doses, indicating no impairment in drug clearance. It is notable that the DMT C_max_ values that we measured after low IN doses in rats (30.1–55.6 ng/mL DMT) are in the same range of DMT concentrations measured in human subjects receiving psychoactive IV doses (Strassman and Qualls 1994; Good et al. 2023; Vogt et al. 2023).

We found that DMT *N*-oxide time-concentration profiles tracked closely with those of DMT after both routes of drug administration, and *N*-oxide concentrations were in the same range as those of the parent compound. Our findings with DMT *N-*oxide in rats differ substantially from results in human subjects receiving DMT, where plasma concentrations of DMT *N-*oxide are far below those of the parent compound (Luethi et al. 2022), suggesting an important species difference between rats and humans in the metabolism of DMT. Prior findings indicate that human tissues generally have higher MAO-A activity when compared to rat tissues (see Inoue et al. 1999), so DMT metabolism in humans might favor the formation of IAA via MAO-A activity rather than formation of DMT *N-*oxide. A key limitation of the present analytical method is the absence of the DMT metabolite, IAA. Future studies should examine the pharmacokinetics of DMT *N-*oxide and IAA in rats treated with DMT.

An additional aim of the present work was to evaluate relationships between circulating concentrations of DMT, DMT *N-*oxide, and pharmacodynamic parameters. Our correlation findings show that mean DMT concentrations from individual rats were positively correlated with mean *N-*oxide concentrations after the IN route but not the SC route. By contrast, mean DMT concentrations were positively correlated with flat body posture scores after both routes, indicating a close relationship between DMT and this behavior after IN or SC administration. With regard to temperature findings, the correlation data were more complex. Specifically, DMT concentrations after SC administration were negatively correlated with body temperature, whereas DMT concentrations after IN administration were not. The lack of correlation between IN DMT and body temperature can be explained by the sustained hypothermic effects after IN dosing, but the reason for such persistent effects of IN DMT is not known. Overall, our findings provide proof-of-principle evidence that DMT can be safely and effectively administered by the IN route in rats, and the plasma concentrations of DMT in rats given IN infusions are similar to those reported in humans receiving psychoactive IV doses (Strassman and Qualls 1994; Good et al. 2023; Vogt et al. 2023). Our data in rats are consistent with recent findings showing IN 5-MeO-DMT can be safely administered to human patients (Rucker et al. 2024), indicating that IN administration might be a viable non-invasive delivery method for examining the acute and sustained effects of DMT in clinical settings.

## Data Availability

All data are available from M.H.B. upon request.
